# Clinical study of different frequency transcranial magnetic stimulation combined with paroxetine in the treatment of poststroke depression with insomnia

**DOI:** 10.1097/MD.0000000000040227

**Published:** 2024-11-08

**Authors:** Xiafei Xu, Liang Li, Tianchu Gao, Qiuping Zhang, Shujuan Liu, Xiyan Liu

**Affiliations:** a Sleep Medicine Department, Hebei Provincial Mental Health Center, The Sixth People’s Hospital of Hebei Province, Baoding, Hebei, People's Republic of China; b Department of Imaging, Cerebrovascular Disease Hospital, Baoding, Hebei, People's Republic of China; c Second Department of Neurology, The First Central Hospital of Baoding City, Baoding, Hebei, People's Republic of China; d Department of Oncology, Mancheng Second Traditional Chinese Medicine Hospital, Baoding, Hebei, People's Republic of China; e Nephrology Department, Traditional Chinese Medicine Hospital, Baoding City, Hebei Province, People's Republic of China.

**Keywords:** insomnia, paroxetine, poststroke depression, repetitive transcranial magnetic stimulation

## Abstract

**Background:**

Study the treatment of poststroke depression and insomnia using varied repetitive transcranial magnetic stimulation (rTMS) frequencies alongside paroxetine. Aim to enhance rTMS effectiveness for depression, insomnia, neurological impairment, and daily living skills.

**Methods:**

Ninety poststroke depression (PSD) patients were randomly divided into a low-frequency group (low-frequency rTMS + enteric-coated paroxetine), a high-frequency group (high-frequency rTMS + enteric-coated paroxetine), and a control group (sham stimulation + enteric-coated paroxetine). The treatment was administered 5 times a week for a total of 2 weeks. Before treatment, at the end of the 2-week treatment, and at the end of the 6-week follow-up, the 3 groups of patients were assessed using the Hamilton Depression Rating Scale (HAMD), Pittsburgh Sleep Quality Index (PSQI), National Institutes of Health Stroke Scale (NIHSS), and Modified Barthel Index (MBI). The mean differences of scores measured at different frequencies and at different times were analyzed by repeated measure one-way analysis of variance.

**Results:**

The interaction between different frequency and score before and after treatment was significant. HAMD, PSQI, and NIHSS scores of the 3 groups after 2 weeks of treatment and 6 weeks of follow-up were significantly lower than before treatment, while MBI scores were opposite. The main effect of treatment scores in different frequency groups showed that HAMD, NIHSS, and MBI scores were not significantly different among the 3 groups before treatment. The HAMD, PSQI, and NIHSS scores of the low frequency group and the high frequency group were significantly lower than those of the control group after treatment, and the MBI scores were the opposite, except that there was no significant difference in HAMD-17 scores among the 3 groups at the 6-week follow-up. Among them, HAMD score in high-frequency group was significantly lower than that in low-frequency group, and PSQI score was significantly higher than that in low-frequency group.

**Conclusion:**

Low-frequency rTMS combined with medication has a better therapeutic effect on the insomnia symptoms of PSD, while high-frequency rTMS combined with medication has a more pronounced therapeutic effect on the depressive symptoms of PSD. Both high-frequency and low-frequency rTMS have an improving effect on neurofunctional deficits and activities of daily living.

## 1. Introduction

Currently, stroke is the leading cause of death in China,^[1]^ with middle-aged and elderly individuals being at high risk for stroke. Poststroke depression (PSD) is one of the most common complications after a stroke, affecting 18% to 33% of stroke survivors according to epidemiological studies.^[2]^ The characteristics of PSD include feelings of sadness, loss of interest, fatigue, decreased self-esteem, difficulty concentrating, impaired decision-making, suicidal thoughts, and other symptoms. These symptoms often persist for an extended duration and may interfere with the social functioning and rehabilitation process of patients.^[2]^ The symptoms of PSD are similar to general depressive symptoms but may interact with stroke-related physical symptoms. Patients may experience physical weakness, fatigue, headaches, and other symptoms, which interact with emotional issues, making depression more prominent^[3]^ PSD may be associated with structural damage to the brain related to stroke, neurotransmitter imbalances, and inflammatory reactions. PSD not only negatively impacts the quality of life and recovery of patients but also increases the risk of suicide.^[4]^ Additionally, it contributes to higher medical costs for stroke patients, as the treatment and care of depressive symptoms require additional resources.

The treatment approaches for PSD typically involve psychotherapy, pharmacotherapy, or a combination of both.^[5]^ Currently, in clinical practice, PSD accompanied by insomnia is primarily treated with a combination of antidepressant drugs and benzodiazepines. However, monotherapy with drugs is associated with significant side effects, potential dependence, especially in elderly PSD patients who are often of advanced age, may have multiple comorbidities, and exhibit low tolerance to medications. During treatment, they are prone to experiencing various side effects, increasing the risks of respiratory suppression, weakness, falls, and bed accidents.^[6]^ Therefore, the exploration of non-pharmacological therapies holds great significance for improving depressive mood, insomnia, the degree of neurological functional impairment, and daily life abilities in this population.

Repetitive transcranial magnetic stimulation (rTMS) is a painless and noninvasive electrophysiological technique.^[7]^ Compared to pharmacotherapy, rTMS does not induce drug-related side effects such as dizziness, nausea, or sexual dysfunction, thereby reducing patient distress. rTMS treatment can selectively adjust the activity of specific brain regions, aiding in the modulation of neural circuits associated with depression and insomnia without causing widespread effects on the entire brain.^[8]^ The benefits of rTMS treatment may persist for an extended period, even after the completion of treatment.^[9,10]^ For patients intolerant or unwilling to use antidepressant drugs, rTMS can serve as an alternative therapeutic option, providing an effective treatment approach.

Despite several studies indicating the efficacy of both high and low-frequency rTMS for PSD,^[11]^ there is no conclusive evidence regarding the comparative effectiveness between high and low-frequency rTMS. Furthermore, research on rTMS for PSD accompanied by insomnia is relatively scarce, with limited studies suggesting the efficacy of low-frequency rTMS,^[12,13]^ and few reports on the use of high-frequency rTMS for treating PSD with insomnia. The adjustment of rTMS treatment parameters, such as stimulation intensity and frequency, based on individual patient conditions and responses, is crucial for optimizing treatment effects. Currently, there is a lack of standardized and rationalized treatment protocols for rTMS, emphasizing the importance of this study, which involves the collection and recording of outpatient and inpatient medical records, to explore the rationalized treatment parameters for rTMS in elderly PSD patients with insomnia.

## 2. Objects and methods

### 2.1. Study subjects

From April 2022 to October 2023, a total of 90 elderly patients with PSD accompanied by insomnia were collected from outpatient and inpatient admissions at our hospital and a local tertiary comprehensive hospital. The patients were sequentially numbered based on the order of inclusion and were randomly divided into low-frequency group, high-frequency group, and control group using a random number table. The study was approved by the Ethics Committee of the Sixth People’s Hospital of Hebei Province. All participants were thoroughly informed about the study and signed informed consent forms.

*Inclusion criteria*: ① All patients aged 45 years or older. ② All patients met the diagnostic criteria for various types of cerebrovascular diseases outlined in the Fourth National Cerebrovascular Disease Academic Conference’s key diagnostic criteria. Diagnosis was confirmed through head CT or brain MRI, indicating either cerebral hemorrhage or cerebral infarction. The onset was the first occurrence, with a stroke duration of 6 months to 2 years. Patients did not exhibit unconsciousness or significant communication barriers and were capable of independently completing relevant scale assessments. ③ PSD patients diagnosed according to the diagnostic criteria for PSD in the 2016 Chinese expert consensus on clinical practice for PSD. Additionally, Hamilton Depression Scale 17-item scores were ≥7. ④ Patients with a Pittsburgh Sleep Quality Index (PSQI) score ≥8 in the past month. ⑤ Patients who cooperated and completed the required examination items during the treatment process, with patients or their family members signing informed consent forms.

*Exclusion criteria*: ① Positive personal or family history of preexisting mental disorders; ② previous or current use of antidepressants or antipsychotic medications. ③ High suicide risk. History of cranial surgery or trauma, personal or family history of epilepsy, comorbid alcohol, or substance dependence. ④ Severe aphasia, hearing impairment, consciousness disorders, and cognitive impairment preventing cooperation with examinations. ⑤ Contraindications to rTMS intervention, such as the presence of a pacemaker, aneurysm clip, intracranial metal implants, etc. ⑥ Severe cardiac, hepatic, renal diseases, severe diabetes, and autoimmune diseases. ⑦ Women in pregnancy, childbirth, or lactation. ⑧ Refusal to sign informed consent forms.

### 2.2. Research methods

#### 2.2.1. Diagnosis and assessment

(1) General assessment

Sociodemographic data include gender, age, occupation, education level, medical history, personal history, family history, smoking, and alcohol habits.

(2) Clinical diagnosis

Clinical diagnosis was made by a deputy chief physician of neurology using the criteria established by the Fourth National Cerebrovascular Disease Academic Conference. Diagnoses were confirmed through head CT or MRI, indicating stroke. Subsequently, 2 attending psychiatrists made diagnoses of depression and insomnia based on the International Classification of Diseases and Related Health Problems, Tenth Edition (ICD-10).

(3) Disease assessment

This includes the assessment of depression severity, sleep disorders, degree of neurological impairment, and daily life activities.

(4) Assessment tools

*HAMD-17*: The HAMD-17 is utilized to reflect the severity of depressive symptoms in patients. Scores range from 0 to 54 points, with scores above 8 indicating potential depression and scores above 17 affirming the presence of depression.^[14]^ *PSQI*: The PSQI is employed to assess the severity of sleep disturbances in the past month. This 19-item scale has a total score of 21, covering 7 factors: sleep quality, sleep onset, sleep duration, sleep efficiency, sleep disturbances, hypnotic medications, and daytime dysfunction. Scores above 7 indicate the presence of sleep disturbances, with higher scores indicating poorer sleep quality.^[15]^ *NIH Stroke Scale* (*NIHSS*): The NIHSS is utilized to evaluate the extent of neurological deficits in patients. Scores range from 0 to 42, with higher scores indicating more severe neurological impairment.^[16]^ *Modified Barthel Index* (*MBI*): The MBI serves as a crucial indicator for assessing the level of independence in patients’ daily activities. With a total of 10 content items, the scale’s score ranges from 0 to 100. Higher scores denote better independence and reduced dependency.^[17]^ *Treatment Emergent Symptom Scale* (*TESS*): The TESS evaluates adverse reactions in patients, encompassing aspects such as dry mouth, constipation, and blood pressure reduction. Higher scores indicate more substantial adverse reactions.

#### 2.2.2. Treatment for the research groups

All 3 groups received secondary prevention of cerebrovascular diseases, self-management rehabilitation training for stroke, sleep hygiene education, and treatment with enteric-coated paroxetine. No form of psychological therapy was administered during the study.

(1) Medication treatment plan

*Basic treatment*: Depending on the type of stroke, secondary prevention of cerebrovascular diseases was administered. Ischemic stroke patients were treated with aspirin and atorvastatin calcium tablets (Aspirin, Bayer Pharma 0.130; Atorvastatin Calcium Tablet 20mg7, Pfizer). For hemorrhagic stroke, symptomatic treatment was provided for risk factors such as hypertension and diabetes, along with specific drug treatments for the underlying disease. All enrolled patients received self-management rehabilitation training for stroke, rehabilitation training guidance, and sleep hygiene education.

*Medication treatment*: Enteric-coated paroxetine 25mg10 (GlaxoSmithKline Pharmaceuticals Limited, imported drug registration number H20170002) was administered orally in the morning at a dose of 25 to 50 mg. Concurrent medication included clonazepam 3mg7 (Chengdu Kanghong Pharmaceutical Group Co., Ltd., National Medical Products Administration approval number H20100074) at 1 to 3 mg per oral intake, used up to 5 times during the trial period.

(2) Treatment for the research group

In addition to the above medication treatment, rTMS was administered. The rTMS treatment method was as follows: The CCY-I type magnetic field stimulator produced by Wuhan Yirede Company was used. All indicators met the requirements of this trial. An 8-shaped coil was selected, and for the low-frequency group, stimulation was applied to the right dorsolateral prefrontal cortex (dlPFC) (F4) at a frequency of 1 Hz, intensity of 90%, stimulation time of 1800 seconds, intermission of 0 seconds, one stimulation, a total of 1800 pulses, and a total treatment time of 1800 seconds. For the high-frequency group, stimulation was applied to the left (dlPFC) (F3) at a frequency of 10 Hz, intensity of 90%, stimulation time of 4 seconds, intermission of 26 seconds, 75 stimulations, a total of 3000 pulses, and a total treatment time of 2250 seconds. Treatment was administered 5 times a week, with 10 sessions constituting one course.

(3) Treatment for the control group

In addition to the above medication treatment, sham rTMS treatment was added. The stimulation coil plane was perpendicular to the patient’s head, forming a sham stimulation by contacting the scalp edge. During treatment, the patient could hear the corresponding frequency “clicking” sound. The treatment duration and course were the same as those for the research group.

#### 2.2.3. Clinical efficacy assessment

Assessments using relevant scales were conducted before treatment, after 2 weeks of treatment, and at the end of the 6-week follow-up. The HAMD-17 was used to assess the degree of depression in PSD patients, the PSQI was used to evaluate patients’ sleep quality, the NIHSS was employed to assess patients’ neurological deficit, the MBI was utilized to evaluate patients’ activities of daily living, and the TESS was employed to assess adverse reactions. Changes in depression, sleep disturbances, neurological deficits, and daily life abilities were compared before treatment, after 2 weeks of treatment, and at the end of the 6-week treatment period, while adverse reaction occurrences were recorded.

### 2.3. Data processing

Statistical analysis was conducted using SPSS 22.0 (IBM Corp., Armonk, NY). Measurement data are presented as mean ± standard deviation (M ± SD). All the data were tested to conform to the normal distribution, and the mean difference of different time points was analyzed by repeated measurement one-way analysis of variance to determine the possible interaction between time points and population. In the process of analysis, all the scores of the 4 groups did not meet the spherical test, so the results of multivariate test should be taken as the standard. *P* < .05 was considered statistically significant. GraphPad software was used for graphical representation. Different uppercase letters and lowercase letters in the figures indicate significant differences between different groups, with different uppercase letters indicating significant differences in scores before and after treatment in the same group, and different lowercase letters indicating significant differences in treatment scores with different frequency parameters.

## 3. Results

Prior to treatment, all participants completed the scale assessments. At the 2-week mark, 4 patients dropped out in the low-frequency group, 5 in the high-frequency group, and 7 in the control group. After treatment, 26 patients in the low-frequency group, 25 in the high-frequency group, and 23 in the control group completed all relevant scale assessments. No significant adverse reactions were observed in all participants, with only 6 reporting transient mild headaches, dizziness, or nausea, which did not interfere with the progress of the experiment.

### 3.1. Comparison of general data among the 3 groups of patients

In the low-frequency group, the age ranged from 45 to 70 years, with an average age of 58.15 ± 11.28 years. There were 14 males and 12 females, with 20 cases of ischemic stroke and 6 cases of hemorrhagic stroke. In the high-frequency group, the age ranged from 46 to 72 years, with an average age of 60.62 ± 11.01 years. There were 14 males and 11 females, with 19 cases of ischemic stroke and 6 cases of hemorrhagic stroke. In the control group, the age ranged from 46 to 74 years, with an average age of 59.68 ± 10.54 years. There were 11 males and 12 females, with 17 cases of ischemic stroke and 6 cases of hemorrhagic stroke.

The 3 groups were comparable in basic data, including age, gender, stroke type, stroke risk factors (hypertension, diabetes, coronary heart disease, smoking, and drinking), education level, recent negative life events, etc (Table 1).

**Table 1:** Comparison of general data among the 3 groups.

Variable	Low-frequency	High-frequency	Control	Statistic	*P*-value
Number of cases (n)	26	25	23	0.465	.865
Age (years of age)	58.15 ± 11.28	60.62 ± 11.01	59.6 ± 10.54	0.437	.603
Male (n, %)	14 (53.8)	14 (56)	11 (47.8)	0.226	.687
Cerebral infarction (n, %)	20 (76.9)	19 (76)	17 (73.9)	0.287	.653
High blood pressure (n, %)	18 (69.2)	17 (68)	15 (65.2)	0.252	.745
Diabetes (n, %)	5 (19.2)	4 (16)	3 (13)	0.083	.611
Coronary heart disease (n, %)	8 (30.8)	7 (28)	7 (30.4)	0.078	.623
Smoke (n, %)	10 (38.4)	12 (48)	11 (47.8)	0.207	.658
Intemperance (n, %)	5 (19.2)	7 (28)	6 (26.1)	1.734	.682
*Schooling* (*n*, %)					
Primary	12 (46.2)	13 (52)	10 (43.5)	0.498	.673
Junior high school	11 (42.3)	10 (40)	11 (47.8)		
Academic	3 (11.5)	2 (8)	2 (8.7)		
Recent negative events (n, %)	5	4	4	0.083	.645

### 3.2. Comparison of HAMD-17 scores before and after treatment in 3 patient groups

**Figure 1.**
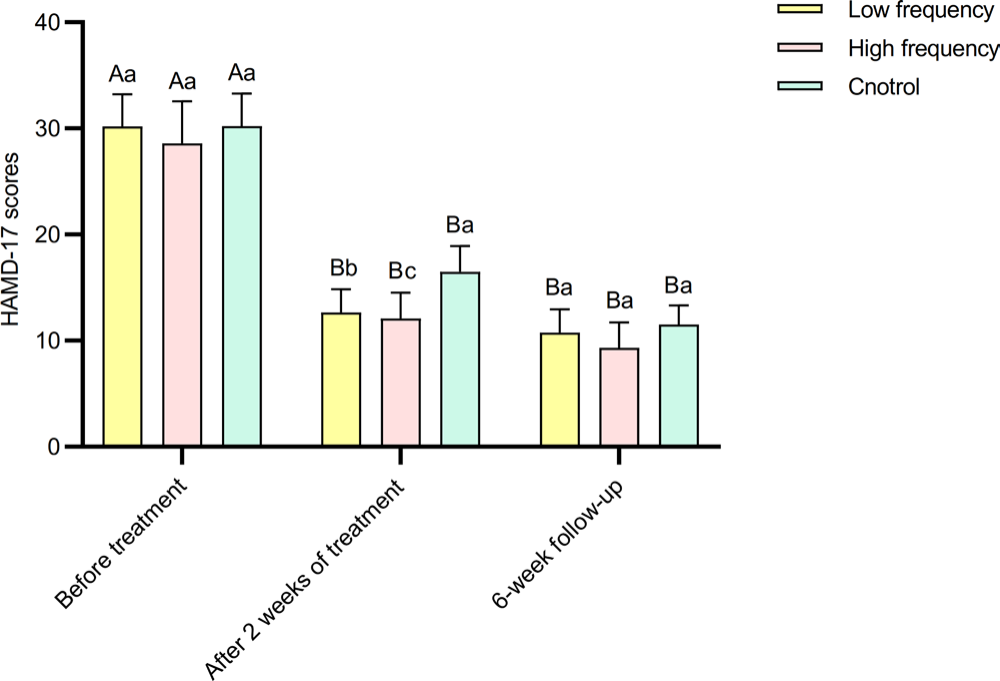
Comparison of HAMD-17 scores before and after treatment in 3 groups. HAMD = Hamilton Depression Rating Scale.

Analysis of variance for repeated measures of HAMD-17 scores before and after treatment in different frequency groups revealed that the main effect of different frequencies was not significant. However, the main effect of measurements before and after treatment was significant (*P* < .01). There was a significant interaction effect between different frequencies and measurements before and after treatment (*P* < .01) (Table 2). With increasing frequency, the HAMD-17 scores gradually decreased after treatment. In the low-frequency group, high-frequency group, and control group, there were significant differences in HAMD-17 scores before and after treatment (*P* < .01) (Table 3). Specifically, the HAMD-17 scores before treatment were significantly higher than those at the 2-week and 6-week follow-ups (*P* < .01) (Fig. 1). Simple effect analysis of groups showed that only at the 2-week follow-up, there was a significant difference in HAMD-17 scores between different frequencies (*P* < .01) (Table 3). The control group’s scores were significantly higher than both the low-frequency and high-frequency groups, and the low-frequency group’s scores were significantly higher than the high-frequency group (Fig. 1) (*P* < .01).

**Table 2:** Repeated measure ANOVA of HAMD-17 scores before and after treatment with different frequencies.

Groups	Prior treatment	After 2 weeks of treatment	Follow-up at 6 weeks
	M ± SD	M ± SD	M ± SD
Low-frequency	29.35 ± 6.72	12.88 ± 3.94	10.31 ± 4.14
High-frequency	28.72 ± 7.87	11.40 ± 4.21	9.28 ± 3.77
Control	29.87 ± 5.82	16.70 ± 4.24	11.83 ± 2.92

	Repeat measurement F test		
	F	*P*	Partial ŋ^[2]^
Main effect of groups	2.588	.085	0.067
The main effect was measured before and after treatment	1135.014	<.001	0.941
Interaction between group and pre - and posttreatment measurements	4.474	.008	0.112

ANOVA = one-way analysis of variance, HAMD = Hamilton Depression Rating Scale.

**Table 3:** Simple effect analysis was performed before and after treatment and in different groups (HAMD-17).

	Groups	Measuring time	F	*P*	Partial ŋ^[2]^
Simple effect analysis measured before and after treatment	Low-frequency	Prior treatment, after 2 weeks of treatment, follow-up at 6 weeks	241.466	<.001	0.083
	High-frequency		251.819	<.001	0.878
	Control		179.819	<.001	0.873

	Measuring time	Groups	F	*P*	Partial ŋ^[2]^
Simple effect analysis of groups	Prior treatment	Low-frequency, high-frequency, control	0.168	.845	0.005
	After 2 weeks of treatment		0.409	<.001	0.227
	Follow-up at 6 weeks		2.907	.061	0.076

HAMD = Hamilton Depression Rating Scale.

### 3.3. Comparison of PSQI scores before and after treatment in the 3 groups of patients

**Figure 2.**
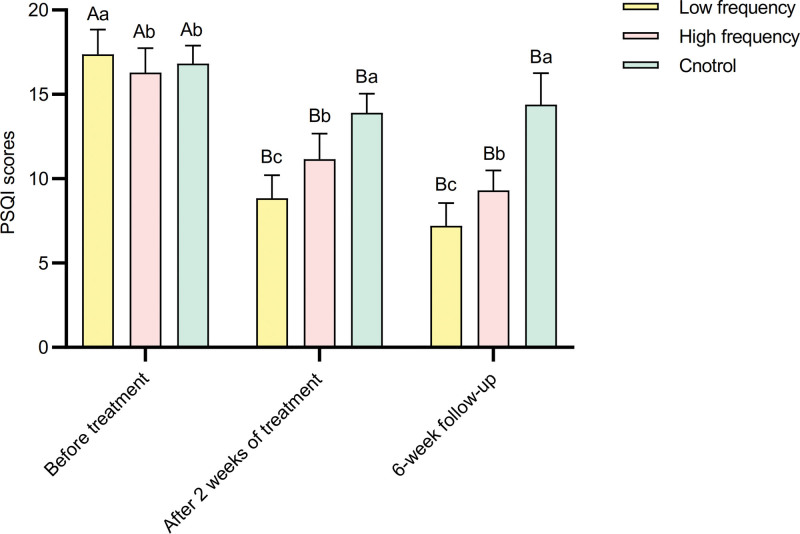
Comparison of PSQI scores before and after treatment in 3 groups. PSQI = Pittsburgh Sleep Quality Index.

Repeated measures analysis of variance for PSQI scores before and after treatment in different frequency groups revealed significant main effects of different frequencies, main effects of measurements before and after treatment, and a significant interaction effect between different frequencies and measurements before and after treatment (*P* < .01) (Table 4). With increasing frequency, the PSQI scores gradually decreased after treatment. The main effects of measurements before and after treatment showed significant differences in PSQI scores among the 3 frequencies before treatment, at 2 weeks, and at the 6-week follow-up (Table 5). In all instances, the PSQI scores before treatment were significantly higher than those at 2 weeks and the 6-week follow-up (*P* < .01) (Fig. 2). Simple effect analysis of groups showed significant differences in PSQI scores between different frequencies before and after treatment. Before treatment, the PSQI scores in the low-frequency group were significantly higher than those in the high-frequency and control groups. At 2 weeks and the 6-week follow-up, the control group’s PSQI scores were significantly higher than those in the low-frequency and high-frequency groups. The high frequency group was significantly higher than the low frequency group (*P* < .01) (Fig. 2).

**Table 4:** Repeated measure ANOVA of PSQI scores before and after treatment with different frequencies.

Groups	Prior treatment	After 2 weeks of treatment	Follow-up at 6 weeks
	M ± SD	M ± SD	M ± SD
Low-frequency	17.23 ± 2.35	8.62 ± 2.33	7.31 ± 2.22
High-frequency	16.96 ± 2.59	10.88 ± 2.42	9.44 ± 2.00
Control	16.83 ± 1.87	14.17 ± 1.97	13.57 ± 3.36

	Repeat measurement F test		
	F	*P*	Partial ŋ^[2]^
Main effect of groups	21.527	<.001	0.377
The main effect was measured before and after treatment	777.366	<.001	0.916
Interaction between group and pre - and posttreatment measurements	63.652	<.001	0.642

ANOVA = one-way analysis of variance, PSQI = Pittsburgh Sleep Quality Index.

**Table 5:** Simple effect analysis was performed before and after treatment and in different groups (PSQI).

	Groups	Measuring time	F	*P*	Partial ŋ^[2]^
Simple effect analysis measured before and after treatment	Low-frequency	Prior treatment, after 2 weeks of treatment, follow-up at 6 weeks	361.592	<.001	0.912
	High-frequency		188.014	<.001	0.843
	Control		32.649	<.001	0.483

	Measuring time	Groups	F	*P*	Partial ŋ^[2]^
Simple effect analysis of groups	Prior treatment	Low-frequency, high-frequency, control	0.918	<.001	0.006
	After 2 weeks of treatment		37.161	<.001	0.511
	Follow-up at 6 weeks		52.033	<.001	0.594

PSQI = Pittsburgh Sleep Quality Index.

### 3.4. Comparison of NIHSS scores before and after treatment in the 3 groups of patients

**Figure 3.**
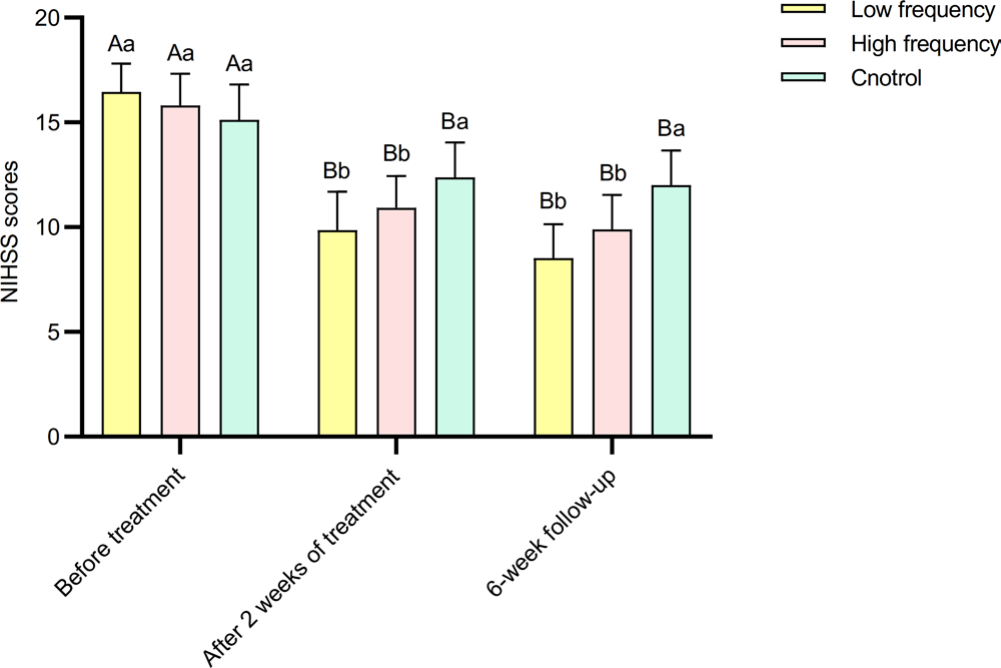
Comparison of NIHSS scores before and after treatment in 3 groups. NIHSS = National Institutes of Health Stroke Scale.

Repeated measures analysis of variance for NIHSS scores before and after treatment in different frequency groups revealed that the main effect of different frequencies was not significant. However, the main effects of measurements before and after treatment were significant (*P* < .01), as well as the significant interaction effect between different frequencies and measurements before and after treatment (*P* < .01) (Table 6). With increasing frequency, the NIHSS scores decreased after treatment compared to before treatment. The main effects of measurements before and after treatment showed significant differences in NIHSS scores among different frequency groups (*P* < .01) (Table 7). Specifically, before treatment, the NIHSS scores in the control group, low-frequency group, and high-frequency group were significantly higher than those at 2 weeks and the 6-week follow-up (*P* < .01) (Fig. 3). Simple effect analysis of groups indicated that only after treatment, there were significant differences in NIHSS scores between different frequency groups (*P* < .01) (Table 7). Specifically, the control group’s NIHSS scores were significantly higher than those in the low-frequency and high-frequency groups (*P* < .01) (Fig. 3).

**Table 6:** Repeated measure ANOVA of NIHSS scores before and after treatment with different frequencies.

Groups	Prior treatment	After 2 weeks of treatment	Follow-up at 6 weeks
	M ± SD	M ± SD	M ± SD
Low-frequency	15.73 ± 2.54	9.50 ± 3.02	8.77 ± 2.78
High-frequency	15.60 ± 2.94	10.8 ± 2.89	10.24 ± 2.80
Control	15.30 ± 2.75	12.22 ± 2.68	11.91 ± 2.60

	Repeat measurement F test		
	F	*P*	Partial ŋ^[2]^
Main effect of groups	2.938	.059	0.076
The main effect was measured before and after treatment	288.73	<.001	0.892
Interaction between group and pre - and posttreatment measurements	8.756	<.001	0.198

ANOVA = one-way analysis of variance, NIHSS = National Institutes of Health Stroke Scale.

**Table 7:** Simple effect analysis was performed before and after treatment and in different groups (NIHSS).

	Groups	Measuring time	F	*P*	Partial ŋ^[2]^
Simple effect analysis measured before and after treatment	Low-frequency	Prior treatment, after 2 weeks of treatment, follow-up at 6 weeks	180.701	<.001	0.838
	High-frequency		102.918	<.001	0.746
	Control		37.046	<.001	0.514

	Measuring time	Groups	F	*P*	Partial ŋ^[2]^
Simple effect analysis of groups	Prior treatment	Low-frequency, high-frequency, control	0.152	.859	0.004
	After 2 weeks of treatment		5.456	.006	0.133
	Follow-up at 6 weeks		8.046	.001	0.185

NIHSS = National Institutes of Health Stroke Scale.

### 3.5. Comparison of MBI scores before and after treatment in the 3 groups of patients

**Figure 4.**
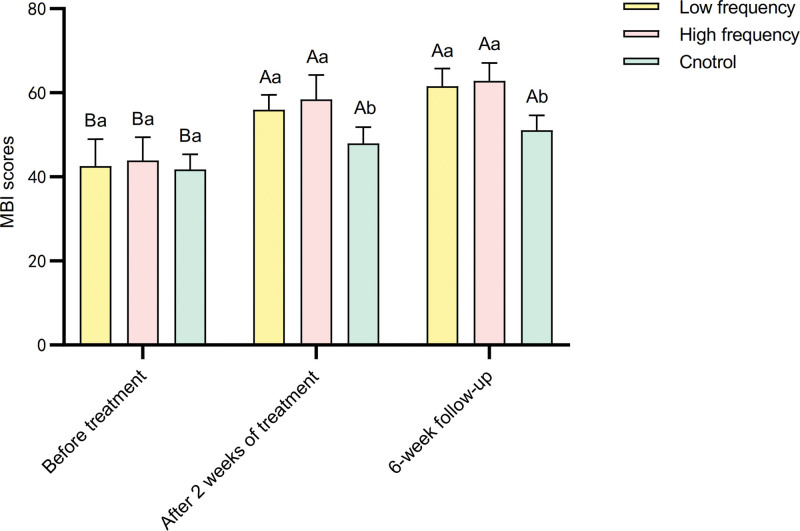
Comparison of MBI scores before and after treatment in 3 groups. MBI = Modified Barthel Index.

Repeated measures analysis of variance for MBI scores before and after treatment in different frequency groups showed significant main effects of different frequencies, main effects of measurements before and after treatment, and a significant interaction effect between different frequencies and measurements before and after treatment (*P* < .01) (Table 8). With increasing frequency, the MBI scores increased after treatment. The main effects of measurements before and after treatment indicated significant differences in MBI scores among different frequency groups (*P* < .01) (Table 9). Specifically, in the control group, low-frequency group, and high-frequency group, the MBI scores at 2 weeks and the 6-week follow-up were significantly higher than those before treatment (*P* < .01) (Fig. 4). Simple effect analysis of groups showed that only after treatment, there were significant differences in MBI scores between different groups (*P* < .01) (Table 9). Specifically, the MBI scores in the low-frequency and high-frequency groups were significantly higher than those in the control group (*P* < .01) (Fig. 4).

**Table 8:** Repeated measure ANOVA of MBI scores before and after treatment with different frequencies.

Groups	Prior treatment	After 2 weeks of treatment	Follow-up at 6 weeks
	M ± SD	M ± SD	M ± SD
Low-frequency	42.30 ± 9.19	56.34 ± 8.07	62.31 ± 6.67
High-frequency	43.00 ± 9.57	57.80 ± 9.14	63.00 ± 8.04
Control	42.39 ± 6.55	48.91 ± 6.73	52.17 ± 6.36

	Repeat measurement F test		
	F	*P*	Partial ŋ^[2]^
Main effect of groups	5.571	.006	0.136
The main effect was measured before and after treatment	415.637	<.001	0.922
Interaction between group and pre - and posttreatment measurements	12.456	<.001	0.264

ANOVA = one-way analysis of variance.

**Table 9:** Simple effect analysis was performed before and after treatment and in different groups (MBI).

	Groups	Measuring time	F	*P*	Partial ŋ^[2]^
Simple effect analysis measured before and after treatment	Low-frequency	Prior treatment, after 2 weeks of treatment, follow-up at 6 weeks	211.484	<0.001	0.858
	High-frequency		209.42	<0.001	0.857
	Control		43.932	<0.001	0.557

	Measuring time	Groups	F	*P*	Partial ŋ^[2]^
Simple effect analysis of groups	Prior treatment	Low-frequency, high-frequency, control	0.048	.953	0.001
	After 2 weeks of treatment		8.272	.001	0.189
	Follow-up at 6 weeks		17.434	.001	0.329

MBI = Modified Barthel Index.

## 4. Discussion

PSD significantly affects the functional recovery and quality of life of patients. Prolonged insomnia after PSD also impacts the cognitive function and neurological recovery of patients. Studies suggest that a decline in daily life activities and cognitive impairment are independent risk factors for PSD, while a lesser degree of neurological impairment is a protective factor.^[5]^ Depression accompanied by insomnia after stroke can worsen the psychological health of patients. Depression and insomnia often mutually influence each other, leading to heightened feelings of sadness, anxiety, and despair in patients. This interplay may disrupt the rehabilitation process for stroke patients.^[18]^ Patients may lack the motivation for rehabilitation treatments such as physical therapy, speech therapy, and occupational therapy due to fatigue and an inability to concentrate. Depression and insomnia negatively impact the quality of life, making patients feel unhappy, fatigued, and socially impaired, thereby reducing life satisfaction.^[19]^ Persistent insomnia and depression may have adverse effects on cardiovascular health, increasing the risks of conditions such as hypertension, heart disease, and stroke. Moreover, it can lead to a decline in cognitive function, including memory difficulties, decision-making problems, and difficulties in focusing attention, making it more challenging for stroke patients to cope with daily life challenges. Depression and insomnia may increase the risk of suicide, particularly in stroke patients.^[20,21]^ Given the severity of PSD with insomnia, it is crucial to diagnose and treat it early to alleviate symptoms, improve the quality of life, and reduce potential harm.

rTMS is a therapeutic approach that involves applying transcranial magnetic stimulation multiple times over a period to influence brain neural activity. This treatment aims to alleviate or improve symptoms associated with neurological and psychiatric disorders by modulating the activity of neuronal networks. rTMS is commonly applied over the prefrontal cortex, an area associated with emotional regulation and the occurrence of depression. By activating this region, rTMS is expected to enhance emotional stability. In recent years, an increasing amount of research indicates that the combination of rTMS and paroxetine significantly improves the treatment outcomes for PSD accompanied by insomnia, demonstrating positive and effective effects.^[22]^ Studies have found that rTMS at different frequencies has a beneficial impact on mental disorders such as poststroke cognitive impairment.^[23]^

This study identified a significant interaction effect between different frequencies and pre-post treatment scores. As the frequency increased, there was a gradual reduction in HAMD-17, PSQI, and NIHSS scores, along with an increase in MBI scores posttreatment. This suggests an improvement in anxiety and depression conditions with different frequency rTMS treatment for PSD. In a study by Yin et al,^[24]^ 20 sessions of 10 Hz rTMS applied to the left dlPFC significantly improved cognitive function and activities of daily living in poststroke cognitive impairment patients. Cognitive improvement was associated with increased low-frequency oscillations in the left dlPFC and enhanced functional connectivity in the right dlPFC and right ventrolateral prefrontal cortex. Different frequencies of rTMS can stimulate specific neural populations in brain regions, leading to changes in various physiological indicators, thereby improving negative states such as depression, anxiety, and insomnia.

Research has shown that high-frequency rTMS is generally considered to have excitatory effects, capable of stimulating neuronal activity in specific brain regions.^[25]^ Chen et al^[26]^ conducted a systematic analysis of the impact of high-frequency rTMS on the daily activities of patients with poststroke cognitive impairment. In 11 randomized controlled trials, the high-frequency rTMS group demonstrated higher scores in Barthel Index, MBI, and functional independence measure compared to the control group. Moreover, the Blessed Behavioral Scale scores in the high-frequency rTMS group were lower than those in the control group. This suggests that high-frequency rTMS can improve the daily activities of patients with poststroke cognitive impairment, yielding favorable rehabilitation outcomes.

The results of this study also indicate that posttreatment patients in the high-frequency group had significantly lower HAMD-17 scores than the low-frequency group, while PSQI scores were significantly higher than the low-frequency group. This implies that the combination of high-frequency rTMS and medication has a more pronounced therapeutic effect on depressive symptoms in PSD whereas the combination of low-frequency rTMS and medication is more effective in addressing insomnia symptoms in PSD. Low-frequency rTMS is considered to have inhibitory effects, slowing down neuronal activity. In certain circumstances, inhibiting excessive activity in specific brain regions may contribute to alleviating depressive and insomnia symptoms. This inhibitory effect might regulate the balance of neural networks, aiding in improving mood and sleep.^[27]^ Low-frequency rTMS may selectively influence specific brain regions, such as the prefrontal cortex, which are closely associated with emotion regulation and sleep control. By modulating these specific regions, interventions can more precisely target the neural circuits associated with PSD.^[28,29]^ Du et al.^[30]^ conducted an analysis of high-frequency and low-frequency rTMS in early stroke patients for motor recovery. The high-frequency rTMS group showed a significant increase in cortical excitability and functional Magnetic Resonance Imaging activation in the ipsilateral motor area, while the low-frequency rTMS group exhibited a significant decrease in cortical excitability and fMRI activation in the contralateral motor area. Both high-frequency rTMS and low-frequency rTMS can improve specific area neural excitability by modulating early stroke motor cortical activation, leading to different improvement outcomes. In conclusion, different frequencies of rTMS yield different effects in treating PSD accompanied by insomnia.

The results of this study show that both low and high frequency rTMS have significant improvement effects on patients with PSD and insomnia, including neurological deficits and activities of daily living. In recent years, several studies have analyzed the efficacy of rTMS in treating PSD patients and related disorders. Fitzsimmons^[8]^ investigated the efficacy and safety of rTMS in obsessive-compulsive disorder, with results showing effectiveness for both dlPFC and medial frontal cortex stimulation. Different unique combinations, including low-frequency supplementary motor area stimulation, high-frequency bilateral dlPFC stimulation, and low-frequency right dlPFC stimulation, were all effective. Lanza^[31]^ studied the treatment of primary sleep disorders with rTMS, revealing subjective improvements with stimulation of bilateral dlPFC, right parietal cortex, and dominant primary motor cortex during insomnia, and stimulation of bilateral M1 leg area, left primary somatosensory cortex, and left M1 during restless leg syndrome. Bai^[32]^ and others suggested that rTMS exhibits anti-inflammatory effects by reducing pro-inflammatory cytokines (including IL-1β, IL-6, and TNF-α) and increasing anti-inflammatory cytokines (including IL-10 and brain-derived neurotrophic factor) in cortical and subcortical tissues. Furthermore, rTMS decreases the expression of nNOS in the same-side dorsal root ganglion and peripheral nerve metabolism, regulating neuroinflammation. In summary, rTMS treatment brings significant benefits to PSD patients.

rTMS is an effective intervention for treating PSD, effectively alleviating depressive symptoms in patients, thereby improving clinical rehabilitation levels and enhancing quality of life. The combination of rTMS with paroxetine treatment has an additive effect on improving depressive symptoms after stroke, and it has a certain improvement on cerebral hemodynamics. However, adjusting stimulation parameters is necessary to achieve neurological benefits. There are differences in the regulatory effects of high and low frequencies on excitability. High frequency is typically used for excitatory regulation, while low frequency is used for inhibitory regulation. rTMS at different frequencies may modulate long-term synaptic inhibition or excitation, affecting synaptic connections between neurons.^[33]^ rTMS can influence neuroplasticity by regulating neurotrophic factors related to neuron survival and function, such as brain-derived neurotrophic factor levels. High-frequency rTMS may impact serotonin levels, thereby exerting a regulatory effect on depressive symptoms.^[34,35]^ rTMS may also modulate the excitability of glutamatergic neurons, influencing overall neuronal excitability. The magnetic field generated by rTMS may induce currents, leading to changes in neuronal membrane potentials, or by modulating functional connections between brain regions, affecting neuronal excitability and the overall activity state of the brain network.^[36]^ This study provides valuable insights into rationalizing treatment parameters for rTMS to improve depressive mood, insomnia, neurological deficits, and daily life activities in PSD patients. It is worth considering the application and promotion of these findings in clinical practice.

The limitations of this study include a relatively small sample size, a short duration of combined rTMS treatment, a short follow-up period, a focus only on differences between different frequencies, and the possibility of bias in the stimulation sites of low-frequency and high-frequency. For various other factors, further clinical research is needed to clarify the optimal treatment modalities.

## Author contributions

**Conceptualization:** Xiafei Xu, Liang Li, Tianchu Gao, Qiuping Zhang, Shujuan Liu.

**Data curation:** Xiafei Xu, Liang Li, Tianchu Gao, Qiuping Zhang, Shujuan Liu, Xiyan Liu.

**Formal analysis:** Xiafei Xu, Liang Li, Tianchu Gao, Qiuping Zhang, Shujuan Liu, Xiyan Liu.

**Funding acquisition:** Xiafei Xu, Liang Li, Tianchu Gao, Qiuping Zhang, Shujuan Liu, Xiyan Liu.

**Investigation:** Xiafei Xu, Liang Li, Tianchu Gao, Qiuping Zhang, Shujuan Liu, Xiyan Liu.

**Methodology:** Xiafei Xu, Liang Li, Tianchu Gao, Qiuping Zhang, Shujuan Liu, Xiyan Liu.

**Project administration:** Xiafei Xu, Liang Li, Tianchu Gao, Qiuping Zhang, Shujuan Liu, Xiyan Liu.

**Resources:** Xiafei Xu, Liang Li, Tianchu Gao, Qiuping Zhang, Shujuan Liu, Xiyan Liu.

**Software:** Xiafei Xu, Liang Li, Tianchu Gao, Qiuping Zhang, Shujuan Liu, Xiyan Liu.

**Supervision:** Xiafei Xu, Liang Li, Tianchu Gao, Qiuping Zhang, Shujuan Liu, Xiyan Liu.

**Validation:** Xiafei Xu, Liang Li, Tianchu Gao, Qiuping Zhang, Shujuan Liu, Xiyan Liu.

**Visualization:** Xiafei Xu, Liang Li, Tianchu Gao, Qiuping Zhang, Shujuan Liu, Xiyan Liu.

**Writing – original draft:** Xiafei Xu, Liang Li, Tianchu Gao, Qiuping Zhang, Shujuan Liu, Xiyan Liu.

**Writing – review & editing:** Xiafei Xu, Liang Li, Tianchu Gao, Qiuping Zhang, Shujuan Liu, Xiyan Liu.
